# HDAC4-Myogenin Axis As an Important Marker of HD-Related Skeletal Muscle Atrophy

**DOI:** 10.1371/journal.pgen.1005021

**Published:** 2015-03-06

**Authors:** Michal Mielcarek, Marta Toczek, Cleo J. L. M. Smeets, Sophie A. Franklin, Marie K. Bondulich, Nelly Jolinon, Thomas Muller, Mhoriam Ahmed, James R. T. Dick, Izabela Piotrowska, Linda Greensmith, Ryszard T. Smolenski, Gillian P. Bates

**Affiliations:** 1 Department of Medical and Molecular Genetics, King’s College London, London, United Kingdom; 2 Department of Biochemistry, Medical University of Gdansk, Gdansk, Poland; 3 Sobell Department of Motor Neuroscience and Movement Disorders and MRC Centre for Neuromuscular Diseases, UCL Institute of Neurology, London, United Kingdom; 4 MRC National Institute for Medical Research, London, United Kingdom; 5 Department of Surgery and Translational Medicine, University of Milano-Bicocca, Milano, Italy; University of Minnesota, UNITED STATES

## Abstract

Skeletal muscle remodelling and contractile dysfunction occur through both acute and chronic disease processes. These include the accumulation of insoluble aggregates of misfolded amyloid proteins that is a pathological feature of Huntington’s disease (HD). While HD has been described primarily as a neurological disease, HD patients’ exhibit pronounced skeletal muscle atrophy. Given that huntingtin is a ubiquitously expressed protein, skeletal muscle fibres may be at risk of a cell autonomous HD-related dysfunction. However the mechanism leading to skeletal muscle abnormalities in the clinical and pre-clinical HD settings remains unknown. To unravel this mechanism, we employed the R6/2 transgenic and *Hdh*Q150 knock-in mouse models of HD. We found that symptomatic animals developed a progressive impairment of the contractile characteristics of the hind limb muscles tibialis anterior (TA) and extensor digitorum longus (EDL), accompanied by a significant loss of motor units in the EDL. In symptomatic animals, these pronounced functional changes were accompanied by an aberrant deregulation of contractile protein transcripts and their up-stream transcriptional regulators. In addition, HD mouse models develop a significant reduction in muscle force, possibly as a result of a deterioration in energy metabolism and decreased oxidation that is accompanied by the re-expression of the HDAC4-DACH2-myogenin axis. These results show that muscle dysfunction is a key pathological feature of HD.

## Author Summary

Huntington’s disease (HD) is a neurodegenerative disorder in which the mutation results in an extra-long tract of glutamines that causes the huntingtin protein to aggregate. It is characterized by neurological symptoms and brain pathology, which is associated with nuclear and cytoplasmic protein aggregates and with transcriptional deregulation. Despite the fact that HD has been recognized principally as a neurological disease, there are multiple studies indicating that peripheral pathologies including cardiac dysfunction and skeletal muscle atrophy, contribute to the overall progression of HD. To unravel the cause of the skeletal muscle dysfunction, we applied a wide range of molecular and physiological methods to the analysis of two well established genetic mouse models of this disease. We found that symptomatic animals developed muscle dysfunction characterised by a change in the contractile characteristics of fast twitch muscles and a decrease in twitch and tetanic force of hindlimb muscles. In addition, there is a significant decrease in the number of motor units innervating the EDL muscle, and this motor unit loss progresses during the course of the disease. These changes were accompanied by the re-expression of contractile transcripts and markers of muscle denervation such as the HDAC4-Dach2-myogenin axis, as well as the apparent deterioration in energy metabolism and decreased oxidation. Therefore, we conclude, that the HD-related skeletal muscle atrophy is accompanied by progressive loss of functional motor units.

## Introduction

Huntington’s disease (HD) is neurodegenerative disorder in which the mutation results in the increased length of a tract of glutamines that causes the huntingtin protein (HTT) to aggregate. It is characterized by neurological symptoms and neurodegeneration that is prominent in the basal ganglia and cerebral cortex [1]. In mammals, HTT is expressed in many tissues and organs [2,3] and is involved in many critical cellular processes such as transcription, protein trafficking and vesicle transport [4]. HTT is predicted to form an elongated superhelical solenoid structure due to a large number of HEAT motifs, suggesting that it plays a scaffolding role for protein complex formation [5]. More than 200 HTT interacting partners have been identified which can be classified according to their function and include proteins that are involved in gene transcription, intracellular signalling, trafficking, endocytosis, and metabolism [6]. In mice, HTT deletion is embryonically lethal, leading to defects in all germ layers [7]. The process of mutant HTT self-aggregation is an early event in HD progression which may lead to the pathological features of HD. Insoluble polyQ aggregates are a hallmark of HD pathology and can be detected at the presymptomatic stage in HD *post mortem* brain [8] and can also be found in many non-central nervous system tissues in HD mouse models [9,10]. Recently, it has been shown that there are a number of factors to indicate that HD patients experience an HD-related heart pathology [11]. A recent study in the R6/2 and *Hdh*Q150 knock-in mouse models showed that the HD-related cardiomyopathy is caused by altered central autonomic pathways and is not due to the accumulation of toxic HTT aggregates as had previously been proposed [12–14]. This was accompanied by the re-expression of foetal genes, apoptotic cardiomyocyte loss and a moderate degree of interstitial fibrosis [13]. There is also growing evidence to indicate that peripheral pathologies such as weight loss and skeletal muscle atrophy may not be a consequence of neurological dysfunction or neurodegeneration and might make a significant contribution to the disease presentation and progression [15]. Therefore it is important to identify the peripheral abnormalities that may contribute to disease progression as they may present targets for new treatment strategies.

To date, our knowledge of skeletal muscle pathology in HD is very limited as outlined in a recent review [16]. A case-study report showed that a semi-professional marathon runner (43 CAGs) developed signs of a slowly progressing myopathy with elevated creatine kinase levels many years before first signs of chorea were detected. A muscle biopsy revealed a mild myopathy with mitochondrial pathology including a complex IV deficiency [17]. Transcriptional deregulation is a typical feature of HD pathology in the brain [18] and a similar transcriptional profile in skeletal muscles (quadriceps) from R6/2 mice, *Hdh*Q150 homozygous knock-in mice and HD patients has been identified that was consistent with a transition from fast-twitch to slow-twitch muscle fiber types [19]. Some of the molecular and physiological changes in HD muscles can even be detected in pre-symptomatic HD individuals [20–22]. At the molecular level, mitochondrial dysfunction, PPAR alpha signalling and HSF1 activation were identified as major players in skeletal muscle HD-related pathology [23,24]. Proof of concept studies have suggested that the progression of disease onset could be delayed and lifespan extended by improving muscle function in HD mouse models [25,26]. However, many aspects of HD neuromuscular transmission and muscle physiology remain unanswered and need to be studied more extensively. In this study, we have investigated the molecular and pathological features of the skeletal muscle dysfunction that develops with disease progression in mouse models of HD at the physiological level.

## Results

To test the hypothesis that mutant HTT leads to the skeletal muscle atrophy through compensatory remodelling, including the HDAC4-myogenin axis, we used two well-established HD mouse models. R6/2 mice are transgenic for a mutated N-terminal exon 1 HTT fragment [27], while the *Hdh*Q150 mice have an expanded CAG repeat knocked-in to the mouse huntingtin gene (*Htt*) [28,29], which is partially mis-spliced with the result that these mice express mutant versions of both an exon 1 HTT and a full length HTT protein [30]. For R6/2 mice, we studied skeletal muscle abnormalities at symptomatic (12 weeks) and end-stage (14 weeks) disease while *Hdh*Q150 homozygotes were compared to wild type (WT) at 22 months (end-stage disease).

**Fig 1:**
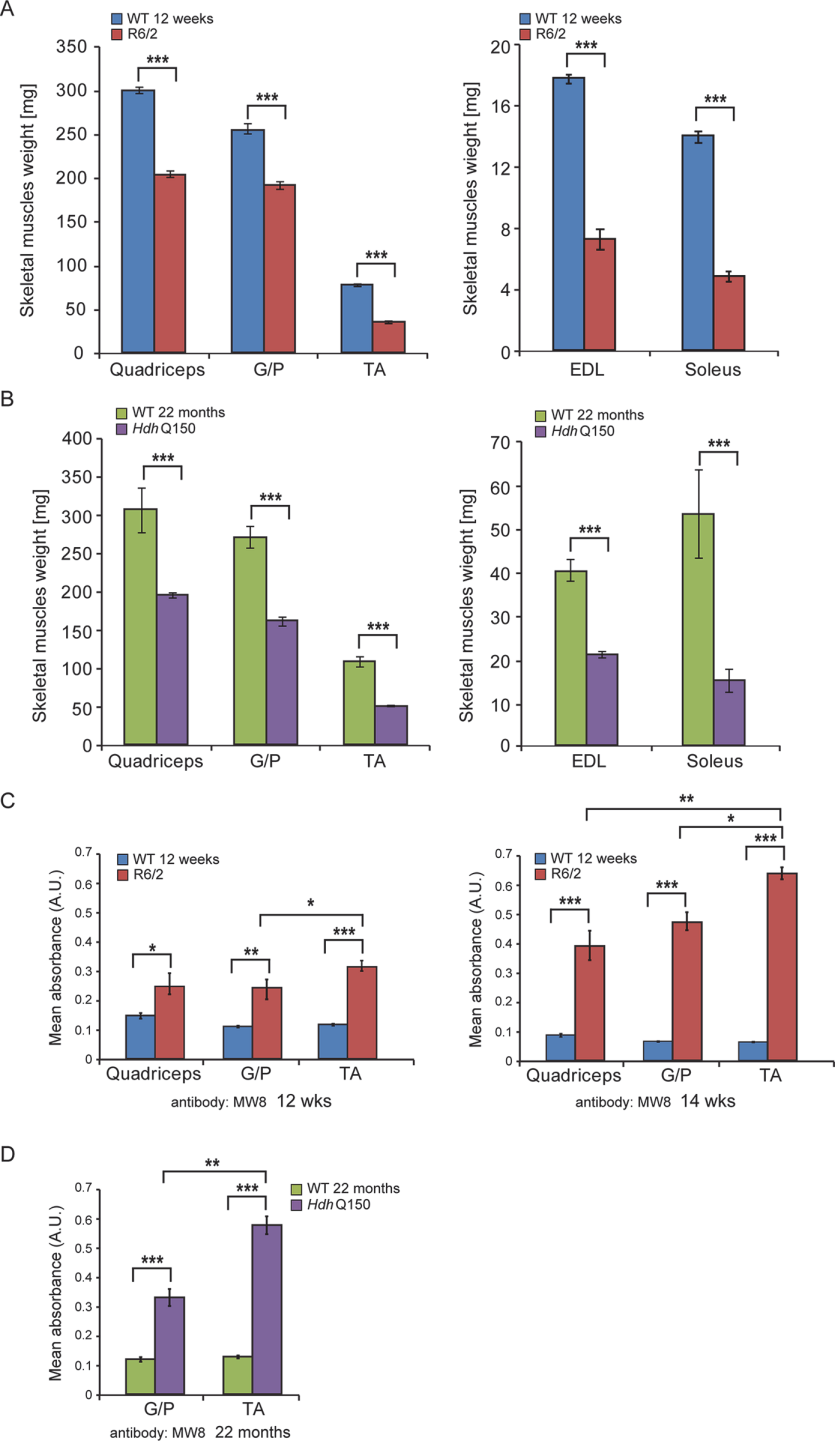
Morphometric and mutant huntingtin aggregation analysis of HD mouse model skeletal muscle. (A) wet skeletal muscle weight of R6/2 mice at 12 weeks of age and (B) *Hdh*Q150 mice at 22 months of age. Quadriceps, gastrocnemius/plantaris complex (G/P), tibialis anterior (TA), extensor digitorum longus (EDL) and soleus. (C, D) The seprion-ligand ELISA was used to identify and quantify the aggregate load in skeletal muscle lysates from R6/2 and *Hdh*Q150 mice. All values are mean ± SEM (*n* = 6/genotype/age). Student’s *t* test: *p < 0.05, **p < 0.01, ***p < 0.001.

We began by quantifying the change in the weight of various skeletal muscles with disease progression (Fig. 1). There was a significant decrease in the muscle mass of all muscles examined types including quadriceps, gastrocnemius/plantaris complex (G/P), tibialis anterior (TA), extensor digitorum longus (EDL) and soleus at 12 weeks of age in R6/2 mice (Fig. 1A). The *Hdh*Q150 knock-in model showed a strikingly similar muscle mass decrease at 22 months of age (Fig. 1B). We have previously shown that HTT inclusions can be detected throughout the periphery of the R6/2 and *Hdh*Q150 mouse models by immunohistochemistry [10,31]. More recently, we have developed the seprion-ligand ELISA, a highly quantitative method with good statistical power that can be used to measure changes in aggregate load that occur *in vivo* in response to pharmacological or genetic manipulations [9]. Using this assay, we were able to detect mutant HTT aggregates in the different skeletal muscle types from either R6/2 at 12 and 14 weeks of age (Fig. 1C) or *Hdh*Q150 (Fig. 1D) mice at 22 months. Surprisingly, we observed a higher accumulation of toxic aggregates in the TA muscles in comparison to G/P and quadriceps in both HD mouse models (Fig. 1C and D). Subsequently, we used Taqman qPCR to demonstrate that the expression of the exon-1 *HTT* mRNA was uniform in the different types of R6/2 skleletal muscles at 12 (S1A Fig.) and 14 weeks of age (S1B Fig.) or *Hdh*Q150 muscle at 22 months (S1C Fig.) and was not therefore the reason for the increased level of aggregates in the TA muscles in both of these models.

**Fig 2:**
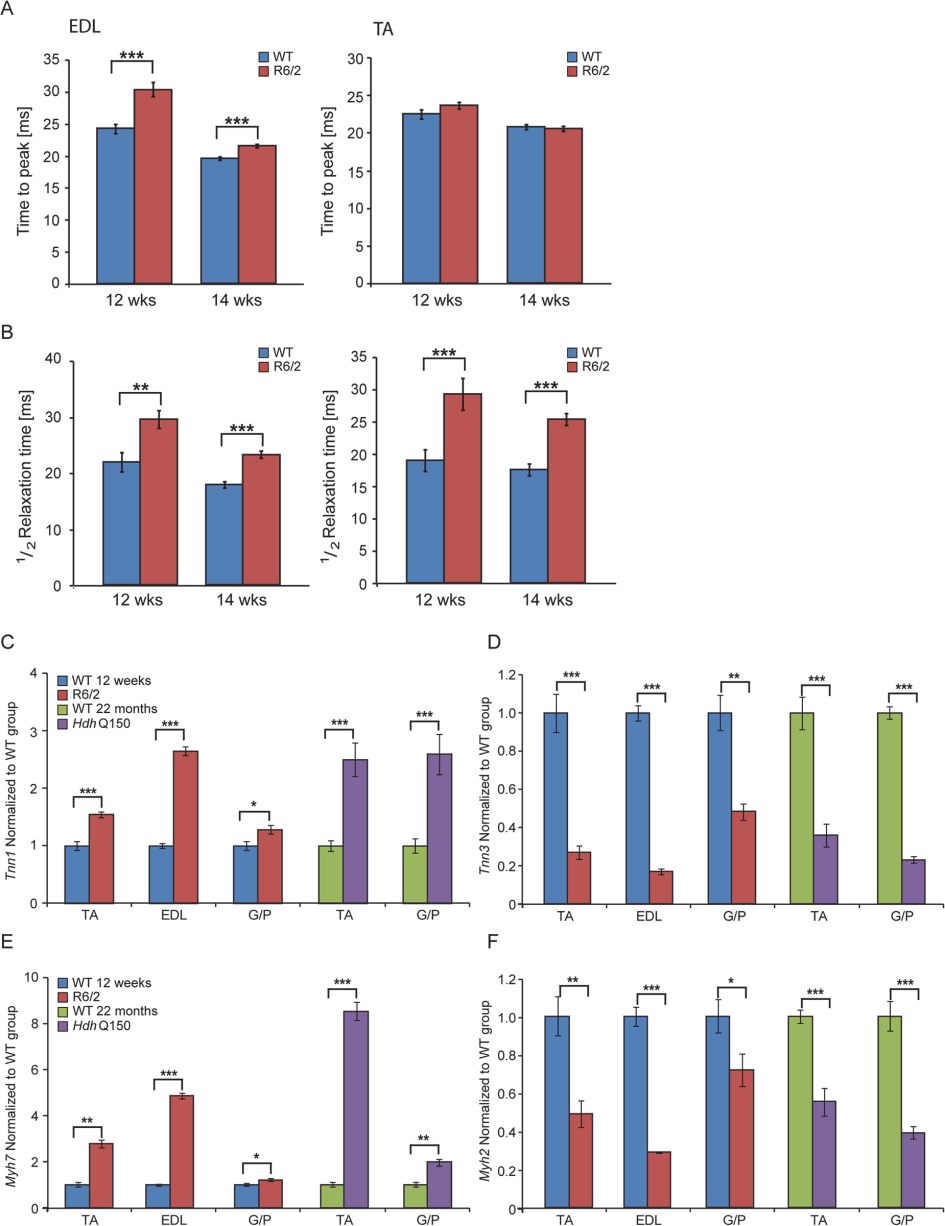
Skeletal muscle contractile dysfunction in the HD mouse models. (A) Twitch stimuli were delivered to elicit TTP (time to peak) contraction of the hind limb EDL and TA skeletal muscles. A significant increase in TTP in the R6/2 skeletal muscles was observed for EDL but not TA at 12 and 14 weeks of age. (B) Twitch stimuli were delivered to elicit a half-relaxation time of the hind limb EDL and TA muscles. A significant increase in the half-relaxation time in R6/2 skeletal muscles was observed for both EDL and TA at 12 and 14 weeks of age. Error bars are SEM (n = 10). ONE-WAY ANOVA with Bonferroni *post-hoc* test: *p < 0.05, **p < 0.01; ***p < 0.001. Transcriptional deregulation of contractile transcripts: (C) *Tnn1* (Troponin 1, slow), (D) *Tnn3* (Troponin3, fast), (E) *Myh7* (myosin heavy light chain 7), (F) *Myh2* (myosin heavy light chain 2) mRNAs were significantly deregulated in the muscle of R6/2 and *Hdh*Q150 mice. All Taqman qPCR values were normalized to the geometric mean of three housekeeping genes: *Atp5b*, *Yhwaz* and *Rpl13a*. Error bars are SEM (n = 6). Student’s *t*-test: *p < 0.05, **p < 0.01; ***p < 0.001.

Next, to determine whether HD mice develop functional contractile abnormalities, we undertook isometric muscle tension experiments on TA and EDL muscles of the R6/2 mouse model and their respective WT littermates, as previously described [32], at 12 and 14 weeks of age. Both of these muscles exhibited a significant degree of atrophy as indicated above (Fig. 1) and significant alterations in their contractile function were revealed (Fig. 2A and B). The time taken for muscles to reach maximum force (time-to-peak, TTP) was significantly higher in the EDL at both time points in R6/2 mice, while TA showed a normal TTP in R6/2 mice in comparison to their WT littermates at both time points (Fig. 2A). The time taken for muscles to relax to a half of the maximum force (1/2 relaxation time) following a twitch-stimulus was significantly prolonged in both EDL and TA muscles in the R6/2 mice at 12 and 14 weeks of age in comparison to their WT littermates (Fig. 2B).

**Fig 3:**
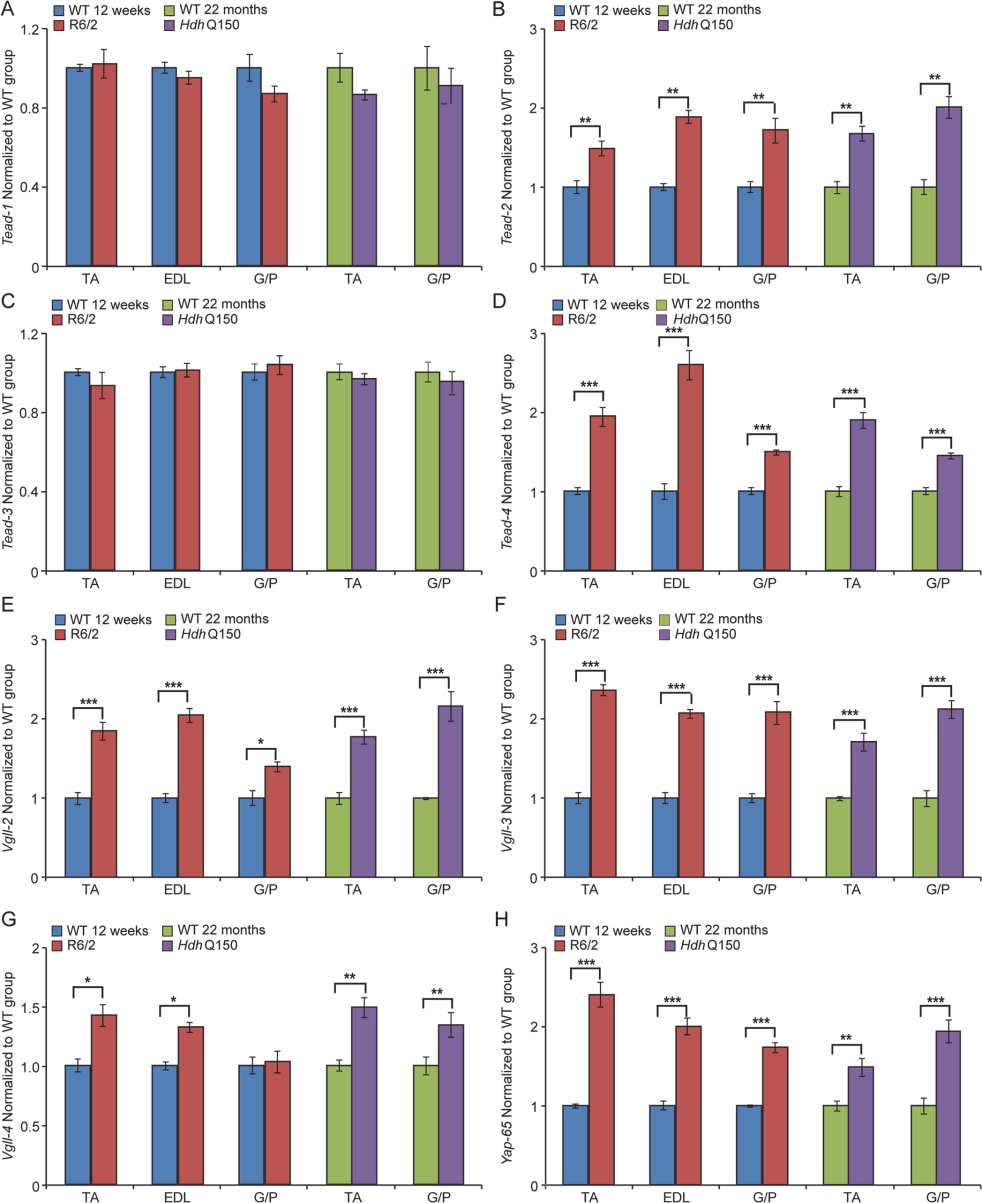
Transcriptional deregulation of TEAD family members and their co-activators involved in the skeletal muscle atrophy. (A) *Tead-1* (TEA domain family member 1), (B) *Tead-2* (TEA domain family member 2), (C) *Tead-3* (TEA domain family member 3), (D) *Tead-4* (TEA domain family member 4). *Tead-2* and *Tead-4* transcripts were deregulated in various muscles of the HD models. (E) *Vgll-2* (vestigial related factor 2), (F) *Vgll-3* (vestigial related factor 3), (G) *Vgll-4* (vestigial related factor 4) and (H) *Yap-65* (Yes associated protein 65) were significantly up-regulated in skeletal muscles of the HD models. All Taqman qPCR values were normalized to the geometric mean of three housekeeping genes: *Atp5b*, *Yhwaz* and *Rpl13a*. Error bars are SEM (n = 6). Student’s *t*-test: *p < 0.05, **p < 0.01; ***p < 0.001.

In order to corroborate the physiological findings described above, we used Taqman qPCR to quantify contractile transcipt levels that are representtive of the fast or slow type fibers. Given that global transcriptional dysregulation is a pathogenic characteristic of HD, we first performed a systematic study to identify suitable reference genes for use in the expression analysis of different skeletal muscles types from HD mouse models. We used the geNorm™ Housekeeping Gene Selection Mouse Kit and associated software to identify the three most stably expressed genes in specific muscles from R6/2 (S2 Fig.) and *Hdh*Q150 (S3 Fig.) mice. Our relative quantification methods then used the geometric mean of these three selected reference genes for normalization, to accurately determine gene expression levels in WT, R6/2 and *Hdh*Q150 skeletal muscle tissue. We found a significant up-regulation of slow-type contractile proteins such as *Tnn1* (Troponin 1, slow) and *Myh7* (myosin heavy light chain 7) in TA, EDL and G/P muscles from both HD mouse models (Fig. 2C and E). Consequently, a pronounced down-regulation of the fast-type contractile proteins like *Tnn3* (Troponin3, fast) and *Myh2* (myosin heavy light chain 2) was also observed in TA, EDL and G/P muscles from both HD mouse models (Fig. 2D and F). These findings indicate that there is a loss of fast-twitch muscle fibres in the EDL and TA of both models. Subsequently, we determined the expression levels of additional genes that are attributed to be altered in fast to slow twitch remodelling. TEA domain (TEAD) transcription factors and their co-activators serve important functional roles during embryonic development as well as in striated muscle gene expression and muscle regeneration [33–36]. It has been shown that striated muscle-restricted TEAD-1 expression induced a transition toward a slow muscle contractile protein phenotype, slower shortening velocity with longer contraction and relaxation times in the adult fast twitch EDL muscles [33]. We found that *Tead-2* (TEA domain family member 2) (Fig. 3B) and *Tead-4* (TEA domain family member 4) (Fig. 3D) were significantly up-regulated in the all diseased HD muscles in both mouse models, while *Tead-1* (TEA domain family member 1) (Fig. 3A) and *Tead-3* (TEA domain family member 3) (Fig. 3C) transcripts remained un-changed. The transcriptional activity of TEAD family members is highly dependent on the presence of their co-activators [37–39] and therefore, we used Taqman-qPCR to asses their transcriptional profile in the HD diseased muscles. We established that *Vgll-2* (vestigial related factor 2) (Fig. 3E), *Vgll-3* (vestigial related factor 3) (Fig. 3F), *Vgll-4* (vestigial related factor 4) (Fig. 3G) and *Yap-65* (Yes associated protein 65) (Fig. 3H) were significantly up-regulated in the TA, EDL and G/P muscles of R6/2 and *Hdh*Q150 mice.

**Fig 4:**
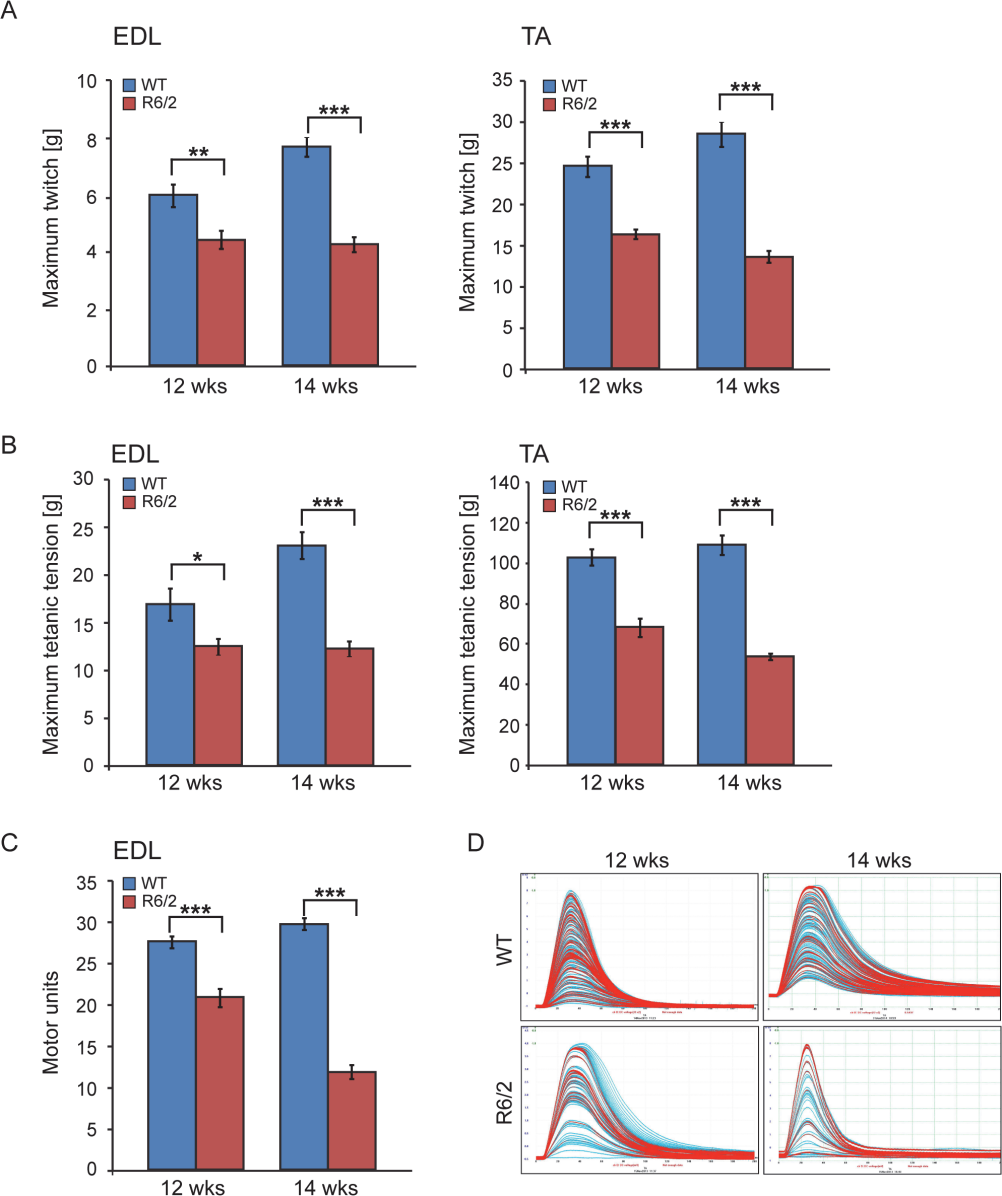
Neuromuscular function in the R6/2 mouse model of HD. (A) A significant decrease in the maximum twitch tension was observed for EDL and TA at 12 and 14 weeks of age. (B) A significant decrease in the maximum tetanic tension was observed for EDL and TA at 12 and 14 weeks of age. (C) The motor unit output for each experimental group is summarised in the bar chart. A significant decrease in functional motor units was observed for EDL at 12 and 14 weeks of age. (D) Examples of the motor unit trace recordings of the WT and R6/2 hind limb EDL muscles at 12 and 14 weeks of age. Error bars are SEM (n = 10). ONE-WAY ANOVA with Bonferroni *post-hoc* test: *p < 0.05, **p < 0.01; ***p < 0.001).

We also determined the maximum muscle force of TA and EDL muscles by physiological determination of single twitch (Fig. 4A) and tetanic force (Fig. 4B) in R6/2 mice. Twitch and tetanic force recordings showed that R6/2 TA muscles at 12 and 14 weeks of age were approximately 50% weaker than in their WT littermates. Moreover, physiological assessment of functional motor unit survival, revealed that there was a significant loss of motor units in R6/2 mice, which progressed from a 25% reduction at 12 weeks to over 60% loss at 14 weeks, compared to WT mice (Fig. 4C and D). These findings suggest that there is also likely to be a significant degeneration of spinal motor neurons during this period.

**Fig 5:**
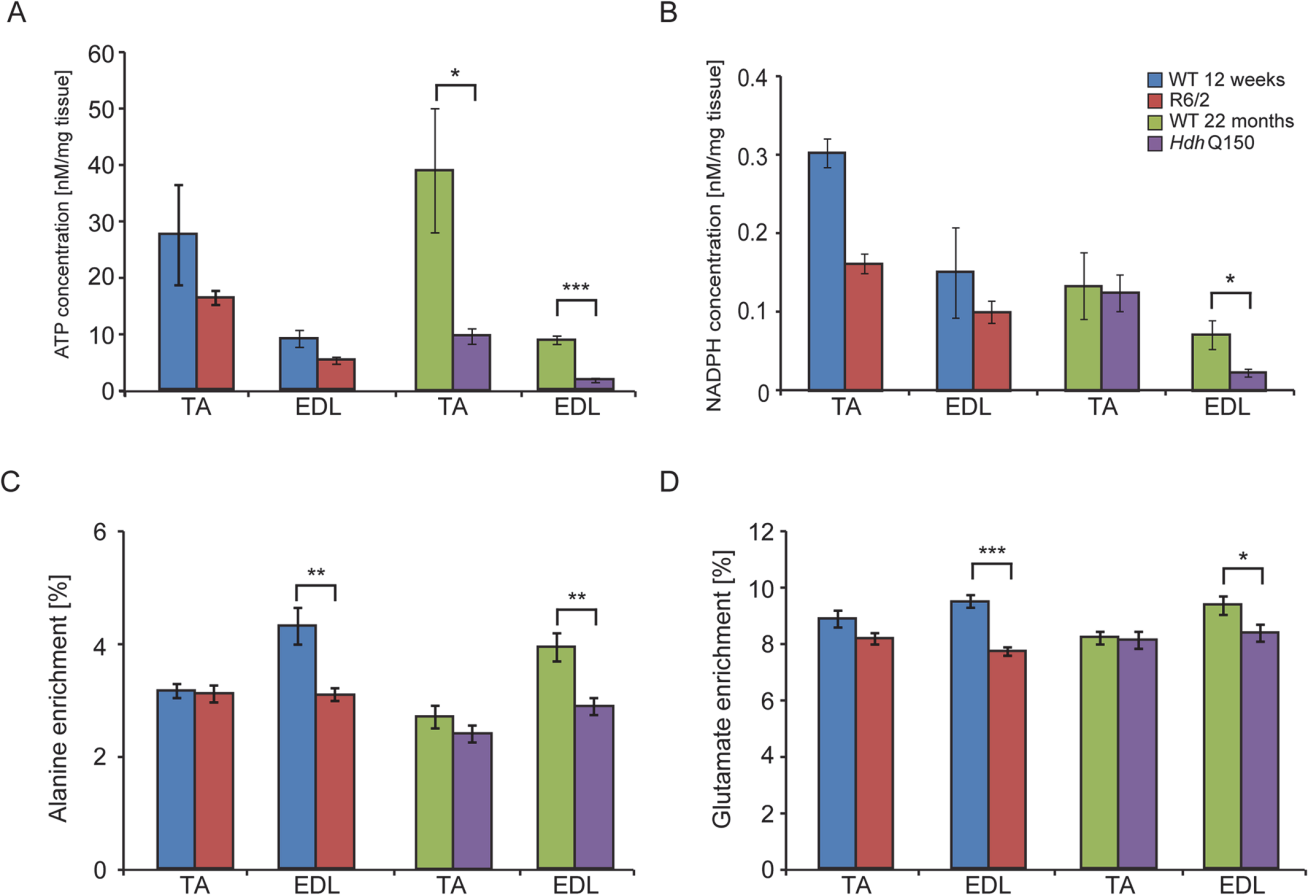
Deterioration of the energy metabolism and decrease in oxidation in the EDL and TA of HD mouse models. Concentrations of (A) ATP (B) NADH (C) alanine and (D) glutamate ^13^C enrichment in skeletal muscle extracts after administration of ^1–13^C glucose in WT and HD mice. Error bars are SEM (n = 6). Student’s *t*-test: *p < 0.05, **p < 0.01; ***p < 0.001.

Physiological changes in skeletal muscle are often caused or associated with metabolic alterations. Therefore, we analysed two aspects of metabolism in the EDL and TA muscles. First we estimated the steady-state concentration of the major components of energy equilibrium that include creatine metabolites and adenine nucleotides. Analysis of ATP, phosphocreatine and related metabolites revealed a substantial depletion of the energy equilibrium in EDL and TA in both HD mouse models (Fig. 5A and B and Table 1). The phosphocreatine/creatine ratio as well as ADP and AMP levels were significantly decreased (Table 1) in both types of muscle in R6/2 and *Hdh*Q150 mice. Besides the energy equilibrium, the total pools of the adenine nucleotides were also consistently depleted (Fig. 5 and Table 1) while changes in the redox status were less evident (Fig. 5B). A similar pattern of metabolic changes was found in the slow type soleus muscles of the HD mouse models (S1 Table). The second metabolic aspect concerned the evaluation of the substrate preference shift in these muscles. To address this, glycolysis was assessed by measuring the ^13^C alanine enrichment while the changes in Krebs cycle were estimated based on ^13^C glutamate levels after administration of ^1–13^C glucose. The analysis revealed that the EDL muscle showed a slower glycolytic flux from exogenous glucose as well less oxidation of glucose in both HD mouse models (Fig. 5C and D) while the TA the muscle remained unchanged (Fig. 5C and D).

**Table 1:** Deterioration of the energy metabolism in the skeletal muscle (EDL and TA) of HD mouse models.

TA				
Measurement/Ratio	WT12 weeks	R6/212 weeks	WT22 months	*Hdh*Q15022 months
ADP [nM/mg tissue]	2.80±0.43	2.54±0.1*	3.56±0.6	2.38±0.2*
AMP [nM/mg tissue]	1.01±0.16	0.94±0.19	1.47±0.23	1.15±0.17
NAD [nM/mg tissue]	2.72±0.52	1.12±0.13**	2.44±0.56	1.44±0.17***
PCr [nM/mg tissue]	219.34±21.29	79.63±5.11***	176.46±28.96	160.92±8.06*
PCr/Cr ratio	5.70±0.57	2.93±0.52**	3.38±0.43	1.95±0.12***
NADH/NAD ratio	0.15±0.02	0.14±0.01	0.06±0.02	0.09±0.01
Total guanine/Total adenine nucleotides ratio	0.039±0.012	0.027±0.006	0.011±0.002	0.027±0.002***
**EDL**				
**Measurement/Ratio**	**WT12 weeks**	**R6/212 weeks**	**WT22 months**	***Hdh*Q15022 months**
ADP [nM/mg tissue]	2.46±0.34	2.42±0.32	2.04±0.17	1.68±0.18*
AMP [nM/mg tissue]	0.22±0.06	0.20±0.05	0.49±0.07	0.37±0.06*
NAD [nM/mg tissue]	1.06±0.20	0.34±0.08**	0.78±0.17	0.21±0.05**
PCr [nM/mg tissue]	157±31.24	50.71±5.74***	52.11±1.97	29.08±1.38***
PCr/Cr ratio	5.78±1.42	2.45±0.32**	2.33±0.11	2.10±0.21*
NADH/NAD ratio	0.10±0.02	0.29±0.01***	0.12±0.02	0.014±0.03
Total guanine/Total adenine nucleotides ratio	0.073±0.011	0.032±0.005**	0.029±0.003	0.013±0.001**

A summary of following parameters are presented: ADP concentration, AMP concentration, NAD concentration, PCr (Phosphocreatine) concentration, PCr/Cr ratio, NADH/NAD ratio, Total guanine to total adenine nucleotides ratio.

**Fig 6:**
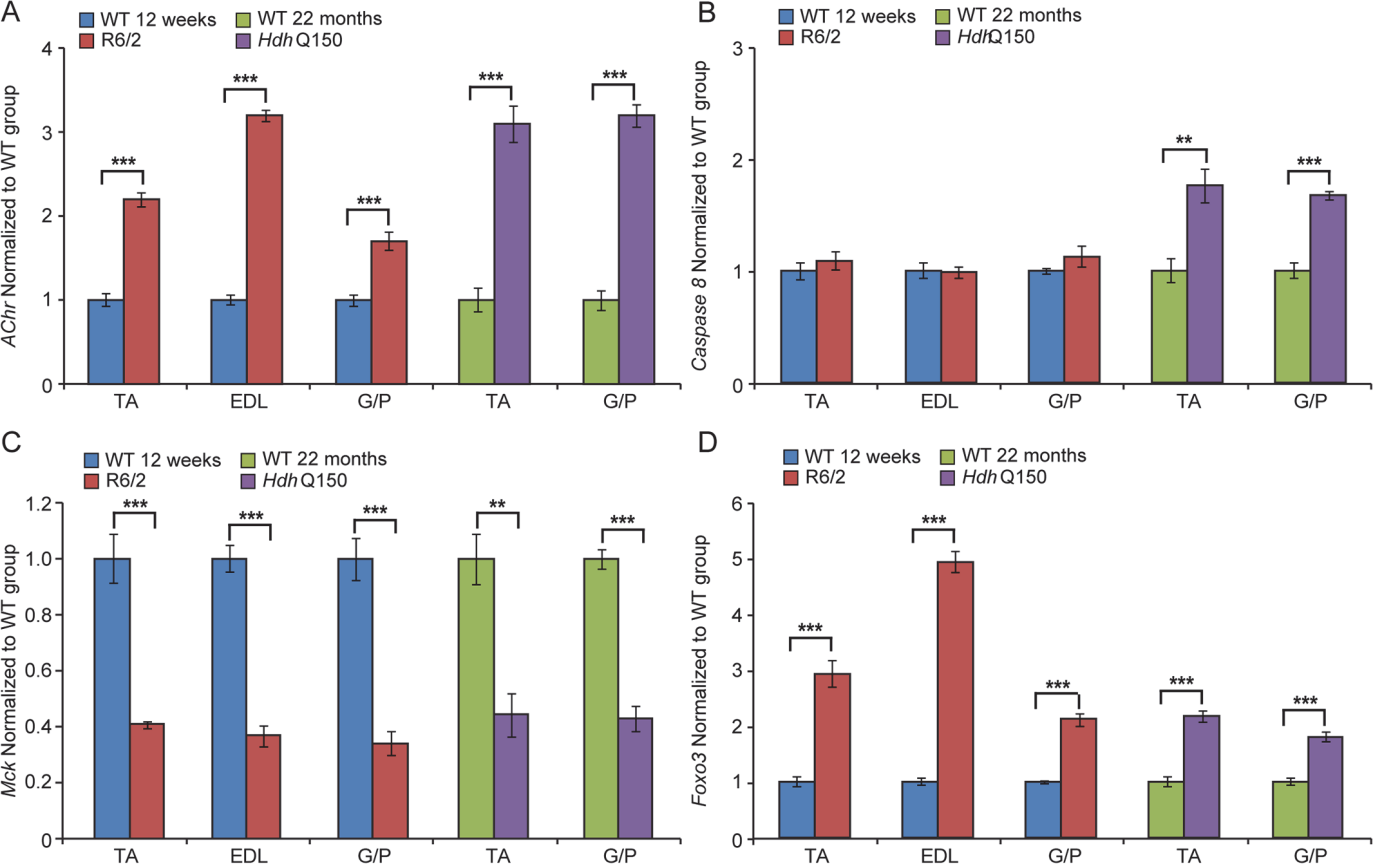
Transcriptional deregulation of gene markers involved in skeletal muscle atrophy. (A) *AChR* (nicotinic acetylcholine receptor), (B) *Caspase8*, (C) *Mck* (muscle creatine kinase) and (D) *Foxo-3* (Forkhead box O3) transcripts were significantly deregulated in various muscles of the HD mouse models. All Taqman qPCR values were normalized to the geometric mean of three housekeeping genes: *Atp5b*, *Yhwaz* and *Rpl13a*. Error bars are SEM (n = 6). Student’s *t*-test: *p < 0.05, **p < 0.01; ***p < 0.001.

To further examine the degree of skeletal muscle pathology, we determined the expression levels of additional genes that are typically altered in atrophied muscles. We found that *AChR* (nicotinic acetylcholine receptor) (Fig. 6A) was significantly up-regulated in all muscle types examined from mouse models. Usually, muscle atrophy is accompanied by a significant up-regulation of caspases [40]. Indeed, we found *Caspase8* transcripts significantly up-regulated in the aged *Hdh*Q150 muscles but not in those from the R6/2 mice (Fig. 6B). Similarly, we found *Foxo-3* (Forkhead box O3) transcripts (Fig. 6D) to be markedly up-regulated, while *Mck* (muscle creatinine kinase) mRNA (Fig. 6C) was decreased in all of the muscle types examines from the R6/2 and *Hdh*Q150 mice.

**Fig 7:**
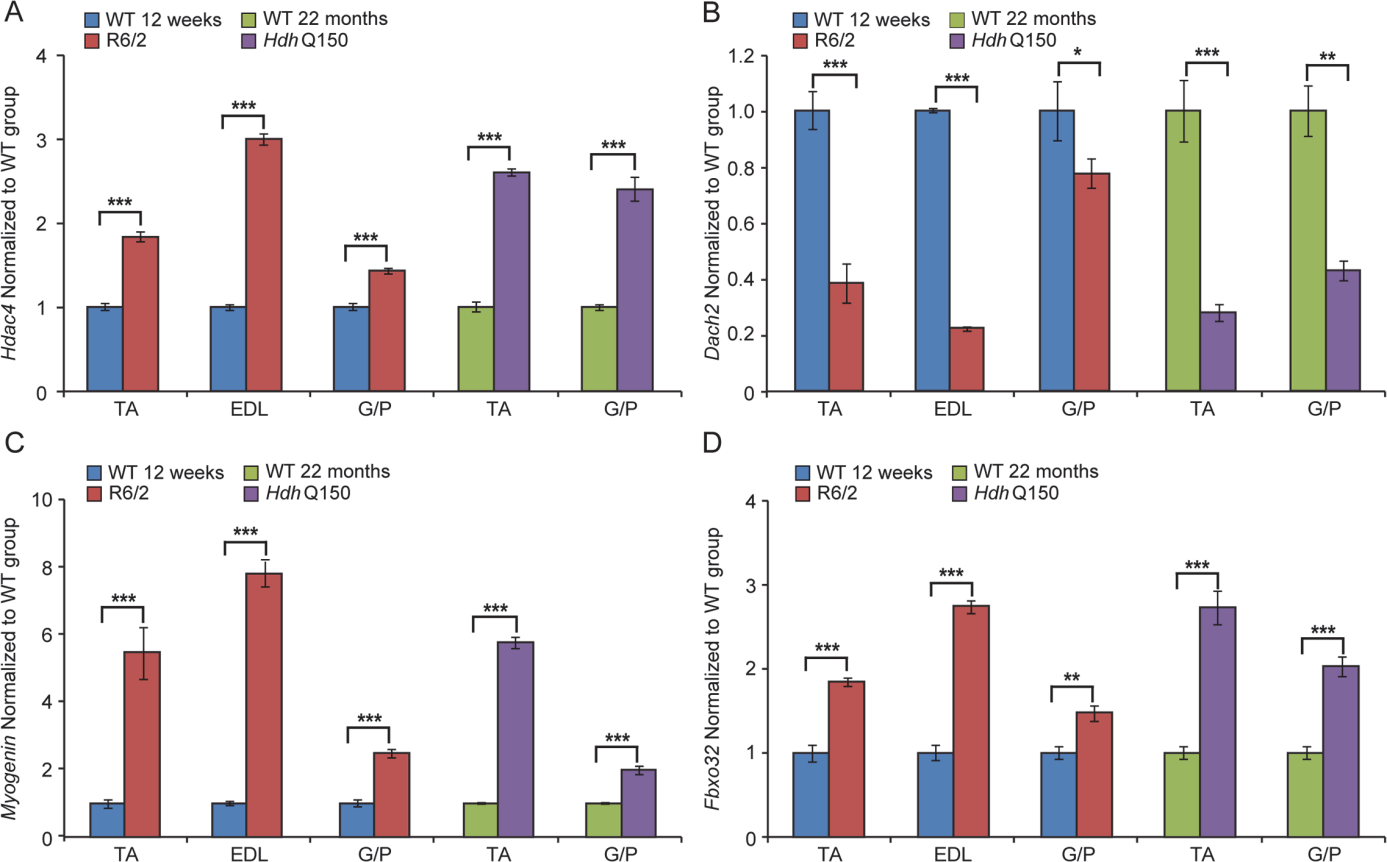
The *Hdac4*-*Myogenin* axis displayed a typical denervation-like phenotype in the skeletal muscle of HD mouse models. (A) *Hdac4* (Histone deacetylase 4), (B) *Dach2* (Dachshund homolog 2), (C) *Myogenin* and (D) *Fbxo32* (F-box only protein 32) mRNAs were significantly deregulated in the skeletal muscles of HD mouse models. All Taqman qPCR values were normalized to the geometric mean of three housekeeping genes: *Atp5b*, *Yhwaz* and *Rpl13a*. Error bars are SEM (n = 6). Student’s *t*-test: *p < 0.05, **p < 0.01; ***p < 0.001.

Previous studies have established HDAC4 as a critical factor that connects neural activity to the muscle remodelling program [41,42] and inactivation of HDAC4 suppressed denervation-induced muscle atrophy while increasing re-innervation [43–45]. HDAC4 up-regulation was found to be significantly greater in patients with rapidly progressive ALS (amyotrophic lateral sclerosis) and was negatively correlated with the extent of muscle re-innervation and functional outcome [46]. Similarly, an increased level of HDAC4 has been found in SMA (spinal muscular atrophy) model mice and in SMA patient muscles [47]. Consistent with this, we found that *Hdac4* transcripts were significantly up-regulated in the TA, EDL an G/P muscles in the HD mouse models as compared to WT littermates (Fig. 7A). *Hdac4* up-regulation was accompanied by down-regulation its direct target *Dach2* (Dachshund homolog 2) (Fig. 7B) that is a negative regulator of *Myogenin*. Consequently, we observed a very significant up-regulation of *Myogenin* (Fig. 7C) and its direct target *Fbxo32* (F-box only protein 32) (Fig. 7D) in HD-related muscle atrophy. Thus, one might conclude that HD-related skeletal muscle atrophy displays the typical characteristics of a denervation like muscle phenotype.

## Discussion

Skeletal muscle is the most abundant tissue in the mammalian body accounting for approximately 40% of body weight, and is composed of multinucleated fibers that contract to generate force and movement. In addition, skeletal muscle possesses a remarkable ability to regenerate, and can go through rapid repair following severe damage caused by exercise, toxins or diseases. The atrophy caused by degeneration of myofibers and their replacement by fibrotic tissue is the major pathological feature in many genetic muscle disorders [48,49]. Skeletal muscle atrophy in HD is a comorbidity that is observed in catabolic disease and other conditions like cancer, congestive heart failure, sepsis, denervation and disuse [16,50]. Under normal physiological conditions muscle function is orchestrated by a network of intrinsic hypertrophic and atrophic signals linked to the functional properties of the motor units that are likely to be imbalanced in HD.

In this study we aimed to provide a broad spectrum of experimental insights into skeletal muscle-associated abnormalities that develop in the R6/2 transgenic and *Hdh*Q150 knock-in HD mouse models, in which mutant *Htt* is expressed under the control of the *Htt* promoter. We found significant alterations at the physiological level in the contractile function of the EDL and TA R6/2 muscles at 12 and 14 weeks of age. The time taken for muscles to reach maximum force (time-to-peak, TTP) and time taken for muscles to relax to half the maximum force (1/2 relaxation time) were significantly changed in R6/2 mice, indicative of a loss of fast-twitch muscle fibres in the EDL and TA muscles. In addition, transcriptional deregulation is a typical feature of HD pathology in the brain [18] and a similar transcriptional profile in the skeletal muscles (quadriceps) from R6/2 mice, *Hdh*Q150 homozygous knock-in mice and HD patients has been identified that was consistent with a transition from fast-twitch to slow-twitch muscle fiber types [19]. Although immunohistochemistry suggested that both type I (slow) and II (fast) muscles were atrophic [31], there were more type I fibers in the R6/2 skeletal muscles. Hence, a conversion of type II to type I fibers has occurred during the process of muscle atrophy [51], most likely as a result of the loss of motor units innervating type II fibres.

Indeed, our physiological findings were also supported by the quantification of contractile transcipt levels that are representative of fast or slow type fibers [52,53]. We found a significant up-regulation of the genes encoding slow-type contractile proteins like *Tnn1* and *Myh7* in the TA, EDL and G/P muscles of both HD mouse models. Consequently, the transcripts for fast-type contractile proteins like *Tnn3* and *Myh2* (myosin heavy light chain 2) were markedly down-regulated in these muscles. This was also accompanied by the up-regulation of members of the TEAD family and their co-activators. It is well established that MCAT elements are located in the promoter-enhancer regions of cardiac, smooth, and skeletal muscle-specific genes and play a key role in the regulation of these genes during muscle development and disease [33–36].

Following a significant decrease in the muscle mass of all of the muscle types that were examined in both HD mouse models, we found that the maximum twitch and tetanic force in TA and EDL hind limb muscles of R6/2 mice were significantly reduced at the symptomatic stage, indicative of motor neuron dysfunction in these mice. Moreover, the physiological assessment of functional motor units revealed that there was a progressive loss in the number of functional fewer motor units in the EDL muscle of R6/2 mice, from ∼25% loss at 12 weeks to more than 60% loss at 14 weeks of age, as compared to their WT littermates. This finding is supported by a previous study showing that skeletal muscles of R6/2 mice developed age-dependent denervation-like abnormalities, including reduced endplate area, supersensitivity to acetylcholine, decreased sensitivity to mu-conotoxin and anode-break action potentials [51]. Moreover, the miniature endplate potential (mEPP) amplitude was notably increased while mEPP frequency was significantly reduced in the R6/2 mice [51]. In contrast, the same study showed that severely affected R6/2 mice developed a progressive increase in the number of motor endplates that fail to respond to nerve stimulation but there was no constitutive sprouting of motor neurons, even in severely atrophic muscles [51]. In fact there was no age-dependent loss of regenerative capacity of motor neurons in R6/2 mice [51]. In line with our findings, a previous study showed that the action potentials in diseased muscles were more easily triggered and prolonged than in WT littermates. Furthermore, the expression of the muscle chloride channel (ClC-1) and *Kcnj2* (Kir2.1 potassium channel) transcripts were significantly reduced and defects in mRNA processing were detected [54]. These dependent denervation-like abnormalities and the highly developed muscle atrophy could be partially explained by sciatic nerve degeneration [55]. A significant decrease in the axoplasm diameter of myelinated neurons and increased number of degenerating myelinated fibers were observed; although the myelin thickness and unmyelinated fiber diameter were not affected [55]. This might be also explained by the profound localisation of mutant HTT to the neuromuscular junctions as was previously published [56]. However, it is likely that the skeletal muscle denervation-like phenotype is linked to not only to spinal motor neuron loss, but also CNS dysfunction, as previously published pathological features in relevant brain regions in both mouse models might support this hypothesis [29]. In HD patient brains, a recent meta-analysis of morphometric MRI found degenerative changes in the amygdala and insular cortex, even in the prodromal form on the disease [57].

It is well established that pronounced skeletal muscles atrophy is accompanied by altered metabolism, reviewed in [58] and our demonstration that the energy equilibrium is depleted in the skeletal muscles of HD mouse models is an important and a novel finding. The decrease in the phosphocreatine/creatine ratio and ATP/ADP ratio directly translates into lower values for phosphorylation potential and the free energy of ATP hydrolysis that might decrease the efficiency of the muscle contraction [52]. Interestingly, our study is in line with previous observations in clinical settings, as muscle ATP/phosphocreatine and inorganic phosphate levels were significantly reduced in both symptomatic and presymptomatic HD subjects [20]. In addition, HD subjects displayed a deficit in mitochondrial oxidative metabolism that might support a role for mitochondrial dysfunction as a key factor involved in the HD-related muscle pathogenesis [21]. An important aspect of this study is the identification of the mechanism underlying the decreased energy equilibrium. One possible explanation is a lack of the trophic effect of nerve stimulation [51,55] that may down-regulate the expression of energy related proteins including factors responsible for mitochondrial biogenesis [58]. Consequently, this process might lead to a decreased oxidative and substrate phosphorylation efficiency translating into a shift of energy equilibrium. Alternatively, a direct local effect of genetic alterations in the skeletal muscle that are likely to be driven by mutant HTT directly [56,59] may deregulate energy metabolism. Interestingly, a similar metabolic profile has been found in mouse embryonic stem cell (mESC) lines: *Htt*(−/−), extended poly-Q (Htt-Q140/7) and wild-type mESCs (Htt-Q7/7) [60]. One might conclude that the HD-related skeletal muscle atrophy is caused by loss of function in HD mouse models.

At the pathological level, the HD-related skeletal muscle atrophy was accompanied by the deregulation of *AChR*, *Foxo-3* and *Mck*, typical markers of muscle atrophy and denervation in both HD mouse models [48,61]. It has been also shown that inactivation of HDAC4 suppresses denervation-like induced muscle atrophy while increasing re-innervation [41,42,45]. These findings highlight a central regulatory role of HDAC4 in activity-dependent muscle remodelling. HDAC4 up-regulation was significantly greater in patients with rapidly progressive ALS (amyotrophic lateral sclerosis) and was negatively correlated with the extent of muscle re-innervation and functional outcome [46]. An increased level of HDAC4 has been found in SMA (spinal muscular atrophy) model mice and in SMA patient muscles [47]. We found an up-regulation of the HDAC4-Dach2-myogenin axis in both HD mouse models that might be indicative of a similar activity dependent muscle remodelling in HD to that observed in ALS or SMA.

**Fig 8:**
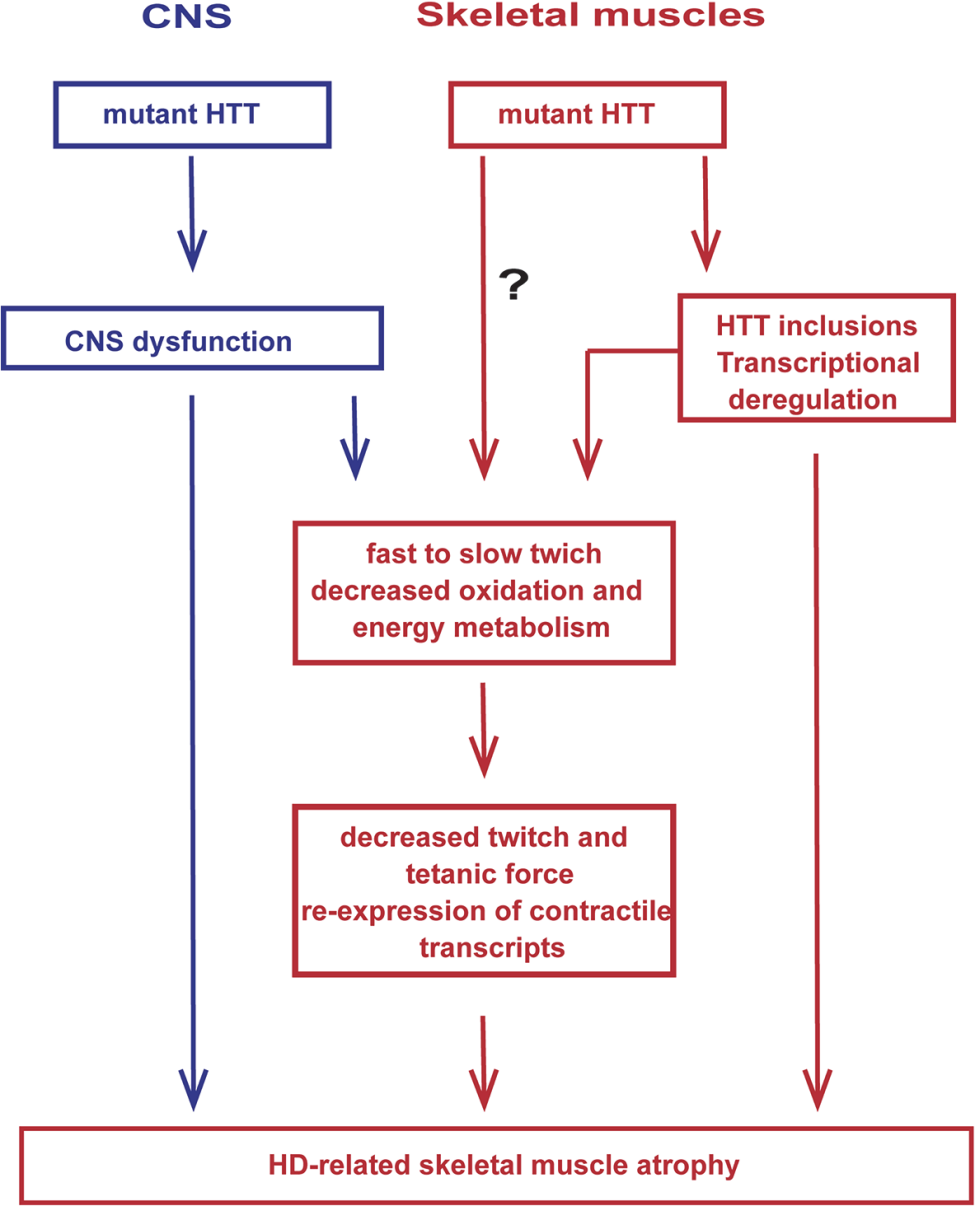
A model depicting the mechanism by which mutant huntingtin causes skeletal muscle wasting in murine HD models. Muscle atrophy is likely driven by the aggregation of mutant HTT in muscle fibres and CNS dysfunction, the relative contribution of which has not been established. It is not possible to rule out that mHTT might exert additional direct detrimental effects within the muscle fibres.

In summary, mutant HTT results in the rapid development of pathological features that would be expected to lead to a skeletal muscle contractile dysfunction e.g. leading to fast to slow fibre twitch with aberrant deregulation of contractile protein transcripts and their up-stream transcriptional regulators. In addition, HD mouse models develop a notable decrease in the twitch and tetanic force of skeletal muscles and pronounced loss of motor units, which may contribute to deterioration of energy metabolism and decreased oxidation that is accompanied by the re-expression of HDAC4-Dach2-myogenin axis (Fig. 8). Importantly, our data connects gene alterations with physiological function in HD-related skeletal muscles atrophy and might have a therapeutic potential. Recently, two key signalling pathways, i.e. those driven by insulin like growth factor (IGF) and growth differentiation factor −8 (GDF-8), have emerged to be potent regulators of skeletal muscle size. In addition, our metabolomic profile of skeletal muscles in HD mouse models might be served as a biomarker platform for prospective pre- and clinical trials.

## Materials and Methods

### Ethics statement

All experimental procedures performed on mice were conducted under a project licence from the Home Office and approved by the King's College London Ethical Review Process Committee.

### Mouse maintenance and breeding

Hemizygous R6/2 mice were bred by backcrossing R6/2 males to (CBA x C57BL/6) F1 females (B6CBAF1/OlaHsd, Harlan Olac, Bicester, UK). *Hdh*Q150 homozygous mice on a (CBA x C57BL/6) F1 background were obtained by intercrossing *Hdh*Q150 heterozygous CBA/Ca and C57BL/6J congenic lines as described previously [29]. All animals had unlimited access to water and breeding chow (Special Diet Services, Witham, UK), and housing conditions and environmental enrichment were as previously described [62]. Mice were subject to a 12-h light/dark cycle. All experimental procedures were performed according to Home Office regulations.

### Genotyping

Genomic DNA was isolated from an ear-punch. R6/2 and *Hdh*Q150 mice were genotyped by PCR and the CAG repeat length was measured as previously described [9] and listed in S2 Table. Dissected tissues were snap frozen in liquid nitrogen and stored at −80°C until further analysis.

### RNA extraction and Taqman real-time PCR expression analysis

Total RNA from skeletal muscles was extracted with the mini-RNA kit according to the manufacturer instructions (Qiagen). The reverse transcription reaction (RT) was performed using MMLV superscript reverse transcriptase (Invitrogen) and random hexamers (Operon) as described elsewhere [63]. The final RT reaction was diluted 10-fold in nuclease free water (Sigma). All Taqman qPCR reactions were performed as described previously [64] using the Chromo4 Real-Time PCR Detector (BioRad). Stable housekeeping genes for qPCR profiling of various skeletal muscles for HD mouse models were determined using the Primer Design *geNorm Housekeeping Gene Selection Mouse Kit with PerfectProbe* software. Estimation of mRNA copy number was determined in triplicate for each RNA sample by comparison to the geometric mean of three endogenous housekeeping genes (Primer Design) as described [65]. Primer and probe sets for genes of interest were purchased from Primer Design or ABI.

### Antibodies, Seprion ELISA

Aggregates were captured in Seprion ligand coated plates (Microsens) and detected using the MW8 mouse monoclonal antibody (1:4000) as described [9].

### Nucleotides, alanine and glutamate concentrations *in vivo*

Mice were injected with glucose-^13^C subcutaneously as a 20% solution at a dose of 3ml/kg. Two hours after glucose administration, mice were deeply anesthetized with isoflurane and EDL and TA muscles were freeze-clamped *in situ* with aluminum clamps pre-cooled in liquid nitrogen. Freeze dried muscles were extracted with 0.4 M perchloric acid, extracts were neutralized with 2 M KOH as described previously [66] and analysed by liquid chromatography mass spectrometry [67] using TSQ Vantage triple quadrupole mass detector linked to Surveyor chromatography system. Mass detection was carried out in fragmentation mode (Tandem MS) and ^13^C isotopic enrichment of fragments containing C3 of alanine or C4 of glutamate were monitored.

### *In vivo* muscle tension

Mice were deeply anesthetized with isoflurane and prepared for *in vivo* analysis of muscle function which was performed as previously described [68]. The distal tendons of the TA and EDL muscles in both hindlimbs were dissected free and attached by silk thread to isometric force transducers (Dynamometer UFI Devices, Welwyn Garden City, UK). The sciatic nerve was exposed and sectioned proximally. The length of the muscles was adjusted for maximum twitch tension. The muscles and nerve were kept moist with saline throughout the recordings and all experiments were carried out at room temperature. Isometric contractions were elicited by stimulating the nerve to TA and EDL using square-wave pulses of 0.02 ms duration at supra-maximal intensity, via silver wire electrodes. Contractions were elicited by trains of stimuli at frequencies of 40, 80 and 100 Hz. The maximum tetanic tension was measured using a computer and appropriate software Pico Technology,Cambridgeshire, UK. The number of motor units innervating the EDL muscles was also determined as previously described [69] by stimulating the motor nerve with stimuli of increasing intensity, resulting in stepwise increments in twitch tension due to successive recruitment of motor axons with increasing stimulus thresholds. The number of stepwise increments was counted to give an estimate of the number of functional motor units (MUNE) present in each muscle. Following recording of isometric tension, the contractile characteristics of EDL and TA muscles were determined. The time to peak (TTP) was calculated by measuring the time taken (ms) for the muscle to elicit peak twitch tension and the half relaxation time (the time taken for the muscle to reach half relaxation from peak contraction) was also calculated. The tetanic contractions were recorded on a Lectromed Multitrace 2 recorder (Lectromed Ltd, UK). All parameters were measured using a computer and Picoscope v5 and v6 software (Pico Technology,Cambridgeshire, UK).

### Statistical analysis

All data were analysed with Microsoft Office Excel and Student's *t*-test (two tailed) or ONE-WAY ANOVA with Bonferroni *post-hoc* test.

## Supporting Information

S1 Fig *HTT* exon-1 transgene levels are stable in the skeletal muscle of R6/2 and *Hdh*Q150 mice. Taqman qPCR showed that *HTT* exon-1 transgene levels are stable in the skeletal muscles of R6/2 (A,B) and *Hdh*Q150 mice (C). The dotted line indicates that the signal in WT animals occurs at the cut-off for gene expression. All Taqman qPCR values were normalized to the housekeeping gene 18S. Error bars are SEM (*n* = 6).
(TIF)

S2 Fig Identification of suitable reference genes for qPCR from skeletal muscle RNA from the R6/2 mouse model. A GeNorm analysis was used to identify optimal reference genes. Raw crossing threshold (C_t_) data for a panel of 12 potential reference genes from the geNorm kit in WT and R6/2 mice (12 weeks old) from (A) TA (B) EDL and (C) G/P. The following gene transcripts were examined: *Atcb* (Actin, beta, cytoplasmic, 11461), *Gapdh* (Glyceraldehydes-3-phosphate dehydrogenase, 14433), *Ubc* (Ubiquitin C, 22190), *B2m*, (Beta-2-microglobulin, 12010), *Ywhaz* (Phospholipase A2, 22631), *Rpl13a* (Ribosomal protein L13a, 22121), *Canx* (Calnexin, 12330), *Cyc1* (Cytochrome c-1, 66445), *Sdha* (Succinate dehydrogenase complex, subunit A, 66945), *18S* (18S rRNA, 19791), *Eif4A2* (Eukaryotic translation initiation factor 4A2, 13682), *Atp5b* (ATP synthase subunit, 11947).
(TIF)

S3 Fig Identification of suitable reference genes for qPCR from skeletal muscle RNA from the *Hdh*Q150 mouse model. A GeNorm analysis was used to identify optimal reference genes. Raw crossing threshold (C_t_) data for a panel of 12 potential reference genes from the geNorm kit in WT and *Hdh*Q150 mice (22 months old) from (A) TA and (B) G/P. The following gene transcripts were examined: *Atcb* (Actin, beta, cytoplasmic, 11461), *Gapdh* (Glyceraldehydes-3-phosphate dehydrogenase, 14433), *Ubc* (Ubiquitin C, 22190), *B2m*, (Beta-2-microglobulin, 12010), *Ywhaz* (Phospholipase A2, 22631), *Rpl13a* (Ribosomal protein L13a, 22121), *Canx* (Calnexin, 12330), *Cyc1* (Cytochrome c-1, 66445), *Sdha* (Succinate dehydrogenase complex, subunit A, 66945), *18S* (18S rRNA, 19791), *Eif4A2* (Eukaryotic translation initiation factor 4A2, 13682), *Atp5b* (ATP synthase subunit, 11947).
(TIF)

S1 Table Deterioration of the energy metabolism in the soleus of HD mouse models. A summary of following parameters are presented: ADP concentration, AMP concentration, NAD concentration, PCr (Phosphocreatine) concentration, PCr/Cr ratio, NADH/NAD ratio, Total guanine to total adenine nucleotides ratio.
(DOCX)

S2 Table Summary of the number of mice per genotype used in all studies and their CAG repeat sizes. SD = standard deviation.
(DOCX)
