# Supplementary figures and images for: HDAC4-Myogenin Axis As an Important Marker of HD-Related Skeletal Muscle Atrophy
Source: PLoS Genet. 2015 Mar 6;11(3):e1005021. doi: 10.1371/journal.pgen.1005021 (PMC4352047; doi:10.1371/journal.pgen.1005021)

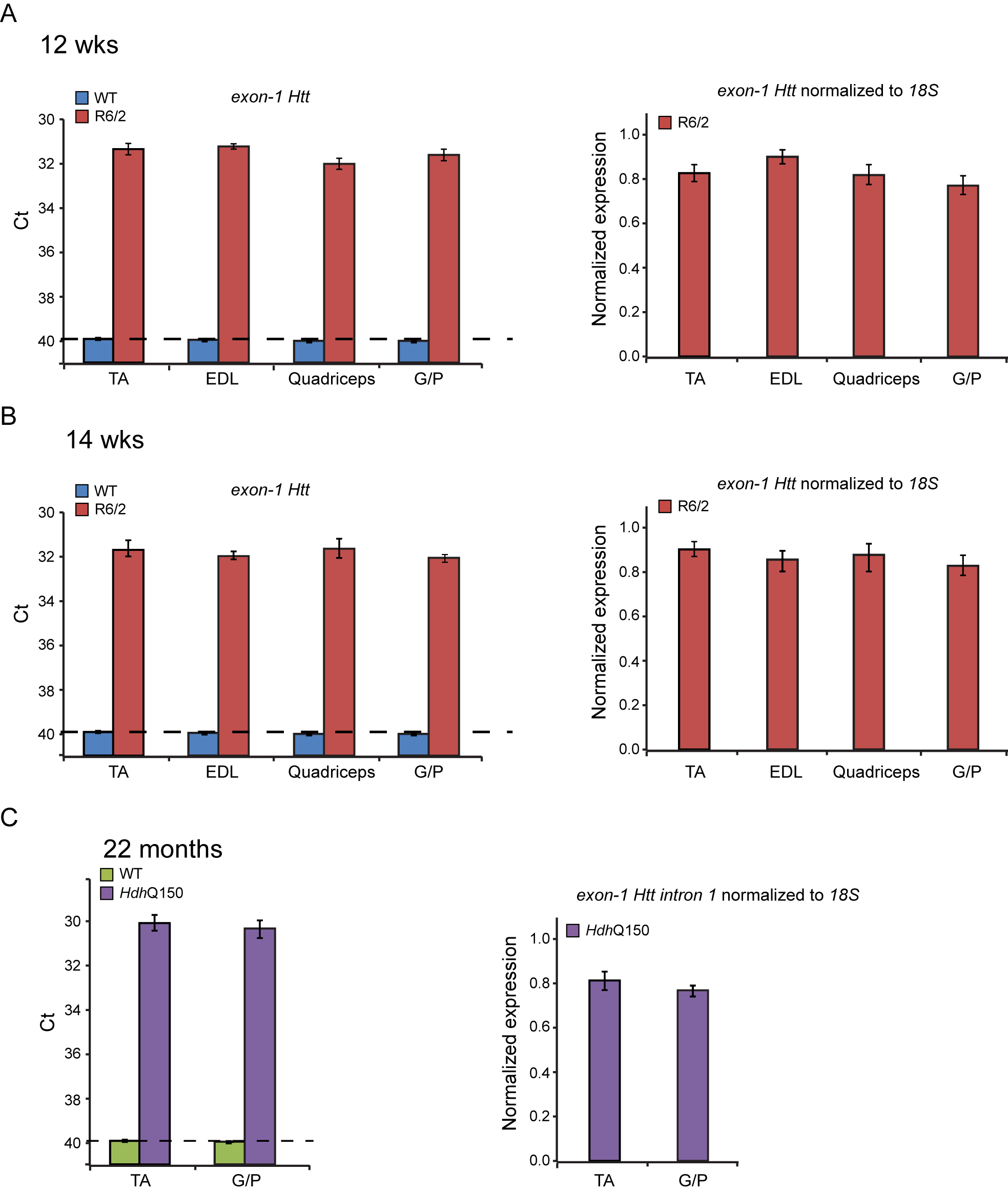

Supplement: S1 Fig — Taqman qPCR showed that HTT exon-1 transgene levels are stable in the skeletal muscles of R6/2 (A,B) and HdhQ150 mice (C). The dotted line indicates that the signal in WT animals occurs at the cut-off for gene expression. All Taqman qPCR values were normalized to the housekeeping gene 18S. Error bars are SEM (n = 6). (TIF) [file pgen.1005021.s001.tif]

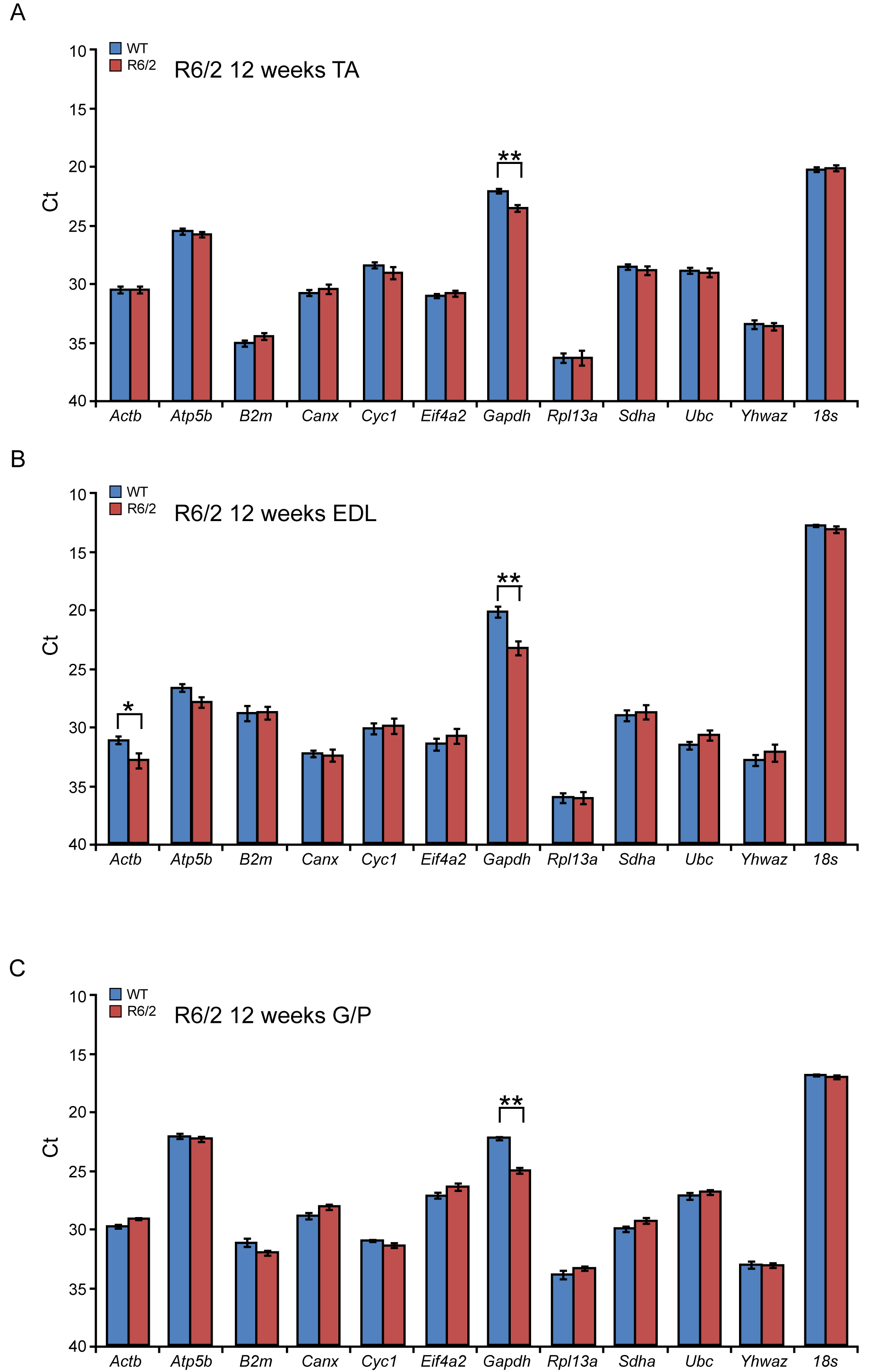

Supplement: S2 Fig — A GeNorm analysis was used to identify optimal reference genes. Raw crossing threshold (Ct) data for a panel of 12 potential reference genes from the geNorm kit in WT and R6/2 mice (12 weeks old) from (A) TA (B) EDL and (C) G/P. The following gene transcripts were examined: Atcb (Actin, beta, cytoplasmic, 11461), Gapdh (Glyceraldehydes-3-phosphate dehydrogenase, 14433), Ubc (Ubiquitin C, 22190), B2m, (Beta-2-microglobulin, 12010), Ywhaz (Phospholipase A2, 22631), Rpl13a (Ribosomal protein L13a, 22121), Canx (Calnexin, 12330), Cyc1 (Cytochrome c-1, 66445), Sdha (Succinate dehydrogenase complex, subunit A, 66945), 18S (18S rRNA, 19791), Eif4A2 (Eukaryotic translation initiation factor 4A2, 13682), Atp5b (ATP synthase subunit, 11947). (TIF) [file pgen.1005021.s002.tif]

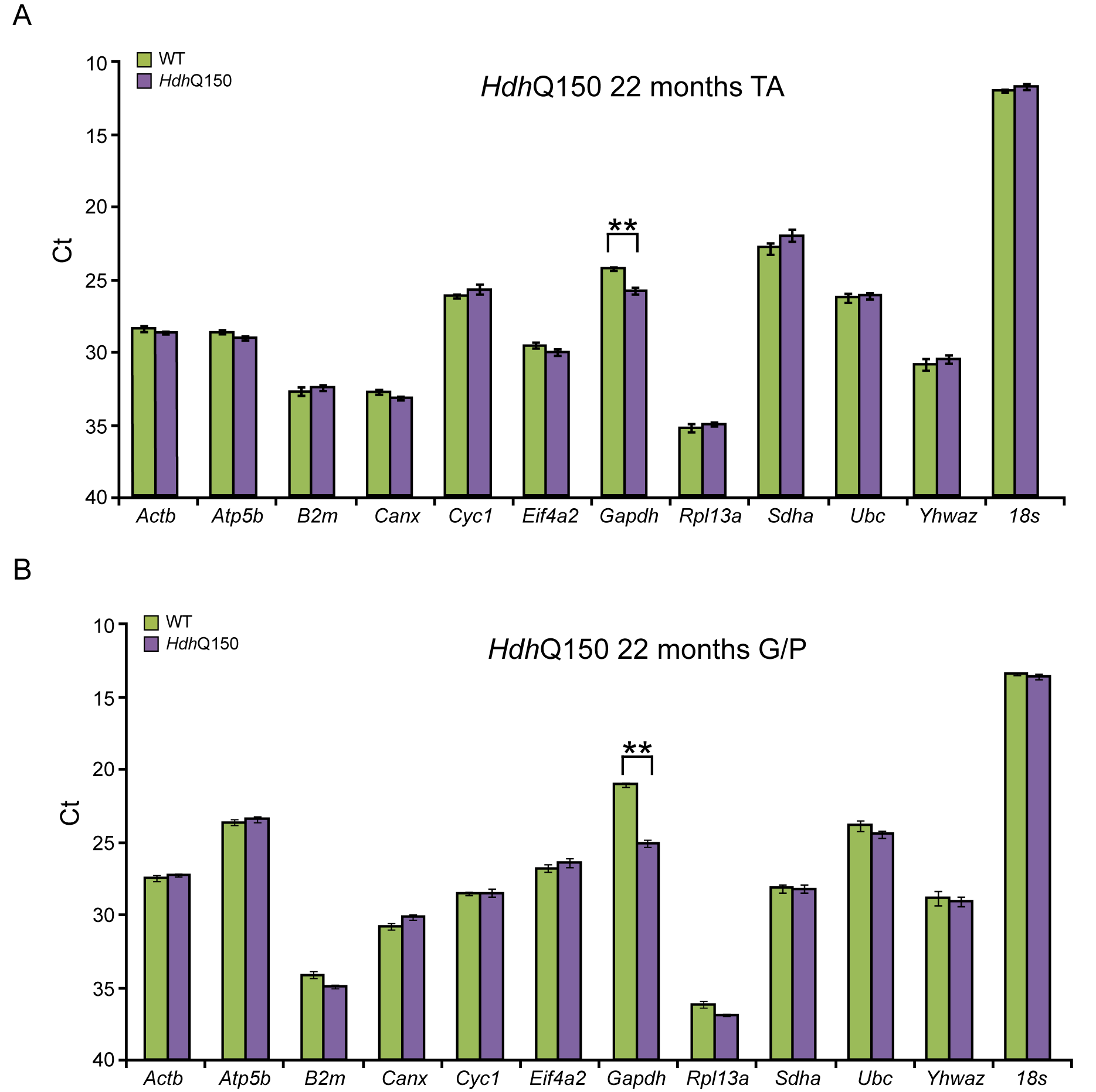

Supplement: S3 Fig — A GeNorm analysis was used to identify optimal reference genes. Raw crossing threshold (Ct) data for a panel of 12 potential reference genes from the geNorm kit in WT and HdhQ150 mice (22 months old) from (A) TA and (B) G/P. The following gene transcripts were examined: Atcb (Actin, beta, cytoplasmic, 11461), Gapdh (Glyceraldehydes-3-phosphate dehydrogenase, 14433), Ubc (Ubiquitin C, 22190), B2m, (Beta-2-microglobulin, 12010), Ywhaz (Phospholipase A2, 22631), Rpl13a (Ribosomal protein L13a, 22121), Canx (Calnexin, 12330), Cyc1 (Cytochrome c-1, 66445), Sdha (Succinate dehydrogenase complex, subunit A, 66945), 18S (18S rRNA, 19791), Eif4A2 (Eukaryotic translation initiation factor 4A2, 13682), Atp5b (ATP synthase subunit, 11947). (TIF) [file pgen.1005021.s003.tif]
