# Supplementary material for: HDAC4-Myogenin Axis As an Important Marker of HD-Related Skeletal Muscle Atrophy
Source: PLoS Genet. 2015 Mar 6;11(3):e1005021. doi: 10.1371/journal.pgen.1005021 (PMC4352047; doi:10.1371/journal.pgen.1005021)
Supplement: S1 Table — A summary of following parameters are presented: ADP concentration, AMP concentration, NAD concentration, PCr (Phosphocreatine) concentration, PCr/Cr ratio, NADH/NAD ratio, Total guanine to total adenine nucleotides ratio. (DOCX) [file pgen.1005021.s004.docx]

**Table S1.**

**Soleus**

| **Measurement/Ratio** | **WT**  **12 weeks** | **R6/2**  **12 weeks** | **WT**  **22 months** | ***Hdh*Q150**  **22 months** |
| --- | --- | --- | --- | --- |
| ADP [nM/mg tissue] | 2.31±0.23 | 2.11±0.31 | 3.11±0.19 | 1.57±0.22*** |
| AMP [nM/mg tissue] | 2.24±0.33 | 0.79±0.17** | 1.79±0.16 | 0.52±0.05*** |
| NAD [nM/mg tissue] | 0.58±0.036 | 0.38±0.038** | 1.00±0.14 | 0.81±0.17 |
| PCr [nM/mg tissue] | 118.98±11.85 | 96.75±12.62 | 144.43±18.53 | 70.31±7.64** |
| PCr/Cr ratio | 7.05±0.75 | 7.07±0.79 | 4.72±0.68 | 4.34±0.87 |
| NADH/NAD ratio | 0.12±0.02 | 0.75±0.01*** | 0.09±0.02 | 0.13±0.01 |
| Total guanine/Total adenine nucleotides ratio | 0.04±0.007 | 0.08±0.01** | 0.06±0.004 | 0.11±0.03* |
