# Supplementary material for: HDAC4-Myogenin Axis As an Important Marker of HD-Related Skeletal Muscle Atrophy
Source: PLoS Genet. 2015 Mar 6;11(3):e1005021. doi: 10.1371/journal.pgen.1005021 (PMC4352047; doi:10.1371/journal.pgen.1005021)
Supplement: S2 Table — SD = standard deviation. (DOCX) [file pgen.1005021.s005.docx]

**Table S2.**

| Study | Genotype | Total number of mice | Mean CAG repeat Size | ±SD |
| --- | --- | --- | --- | --- |
| *In vivo* skeletal muscle tension | WT | 12 | - | - |
|  | R6/2 | 12 | 207 | 8.9 |
| Metabolic study | WT | 6 | - | - |
|  | R6/2 | 6 | 207 | 4.5 |
|  | WT | 6 | - | - |
|  | *Hdh*Q150 | 5 | 166/188 | 2.9/7.5 |
| Molecular biology tests  R6/2 and *Hdh*Q150 mice  Various studies: 12 and 14 weeks and 22 months respectively | WT | 40 | - | - |
|  | R6/2 | 40 | 206 | 7.5 |
|  | WT | 18 | - | - |
|  | *Hdh*Q150 | 18 | 161/191 | 8.5/6.2 |

SD = standard deviation
